# Functional Nervous Disorders in Childhood

**Published:** 1910-03

**Authors:** 


**Functional Nervous Disorders in Childhood.** By LEONARD G. GUTHRIE, M.A., M.D., F.R.C.P. Pp. x, 300. London: Oxford Medical Publication. 1907.

This book is a rarity among modern medical books. Not only is it a thoroughly practical manual of the subject with which it deals, but it is written in a style which carries the reader along pleasantly and profitably to the last page. It is written by a clinician sitting in the library arm-chair, and the delightful blend of a wide reading in general literature with the practical detail of medical treatment may be illustrated well by the following extracts from the index, thus : " Pepys ; Petit mal ; Phillpotts "—Eden, by the way ; " Phimosis, as a cause of eneuresis, of stammering ; Pianese ; Pica ; Pitres ; Plato ; Poe ; Polyuria ; Porridge ; Porot, A. ; Poynton and Paine ; Psammetichus ; Puberty, splenic pain in." And again : " Tertullian ; Tetany ; Thackeray ; Theresa of Castile ; Thigh rubbing ; Thompson (*sic*), John ; Thymic asthma ; Tics ; Tissot ; Tobacco ; Tolstoy ; Traumatism, as a cause of neurasthenia ; Triboulet ; Trollope, Anthony."

In dealing with these neurotic children, and incidentally with their parents, there is shown throughout a healthy common sense, tempered with sympathy. The author's views are clearly expressed without too much dogmatism, and his sense of humour, keen but kindly, is a delight to the reader.

Such chapters as those on " Mental and Educational Overstrain," " Moral Failings of Nervous Children," are particularly valuable, for the family physician is frequently consulted on these matters, and it is difficult to find a book where they are treated in any way adequately.

The chapter on chorea contains the author's method of treating this disease. He does not rely entirely upon rest and drug treatment, but by means of suggestion and guided movements tries to restore the complete control of the will over voluntary movements.

Some of the diseases included in this volume cause at first some surprise at their inclusion, such conditions as idioglossia, hypertrophic stenosis of the pylorus, and migraine. The last disease, common in children and frequently overlooked, seems to occur rather more frequently amongst neurotic children, but not to any very striking extent; and there is a strange omission of any reference to the use of podophyllin in sub-purgative doses, a drug as useful in this disease in children as it is in adults.

It is most necessary that those who have much to do with schools, both teachers and physicians, should thoroughly understand the underlying temperament of the pupils, and realise that when the neurotic temperaments are understood some of the most brilliant successes will be found amongst these neurotic children.

Although the title of this book has obviously not been chosen with that end in view, the book has already found its way on to the shelves of many teachers and parents of nervous children, and the practitioner will be wise if he provides himself with a copy of this sound and useful work.

